# One Health and Hansen’s disease in Brazil

**DOI:** 10.1371/journal.pntd.0009398

**Published:** 2021-05-27

**Authors:** Patrícia Deps, Patrícia S. Rosa

**Affiliations:** 1 Department of Social Medicine and Postgraduate Programme in Infectious Diseases, Federal University of Espírito Santo, Vitória, Espírito Santo, Brazil; 2 Division of Research and Education, Instituto Lauro de Souza Lima, Bauru, São Paulo, Brazil; Colorado State University - Global Campus, UNITED STATES

## One Health and Hansen’s disease

The concept of One Health originated with the German pathologist Rudolf Virchow in the late 19th century but has become a global movement only in the past 2 decades [1]. One Health recognizes that issues of human health and well-being cannot be addressed in isolation but must be considered in the context of equilibrium between all living things and the natural environment [2]. These aspects of people’s health have become more important with human population growth, climate change, ecological pressures, and globalization [3].

Zoonoses present one of the most obvious One Health challenges [4]. In 1971, researchers in the United States of America reported that armadillos reproduce systemic and neurocutaneous forms of leprosy if inoculated with *Mycobacterium leprae* [5]. Natural infection of an armadillo of the species *Dasypus novemcinctus* was reported 6 years later, and sporadic autochthonous cases have since been identified in which the persons affected reported contact with armadillos but no other risk factors [6]. Hansen’s disease is now officially recognized as a zoonosis in the USA, based on evidence of widespread natural infection of armadillos with *M*. *leprae* [7] and evidence of Hansen’s disease arising from direct and indirect contact with wild armadillos [8,9].

In Brazil, almost 30,000 new cases of Hansen’s disease are diagnosed each year, with some areas experiencing hyperendemicity (≥40 cases/100,000 population per year) [10]. Brazil is within the range of several armadillo species which inhabit the Amazon, Atlantic Forest, and semiarid ecosystems [11]. Natural infection of wild armadillos by *M*. *leprae* has been reported across Brazil, equivalent to 1 in 10 animals being infected with the bacillus [12]. All armadillo species are protected in Brazil, and some are critically endangered [13], but their habitats are dwindling in size and disturbed by human activity, and illegal hunting to obtain meat for private consumption and commercial sale is widespread [11]. These activities result in frequent direct and indirect contact between humans and potentially infected armadillos [14], but zoonotic transmission of *M*. *leprae* is currently disregarded in Brazil as an issue for Hansen’s disease control and prevention.

The suspicion that Hansen’s disease might not only affect humans dates to the early 20th century when contagion through direct contact with a person infected by the recently discovered *M*. *leprae* bacillus had become the predominant belief. This gave rise to sanatoriums or “colonies” as a coordinated public health response. Nonetheless, at the Second International Leprosy Congress in 1909, it was suggested that *M*. *leprae* might also exist in the environment (soil and plants) and in animals [15].

The environmental hypothesis has been borne out by studies based on plants, soil, and water sampled in non-endemic and endemic countries [16], including viable *M*. *leprae* detected in soil near the homes of persons affected by Hansen’s disease in India [17]. Natural infection of animals with *M*. *leprae* has been reported for several species [16], including 2 recent chimpanzee cases in West Africa [18]. However, the primary focus on Hansen’s disease as a zoonosis revolves around armadillos in the Americas.

Armadillos belong to the superorder of placental mammals, Xenarthra, inhabitants of the Americas for 60 million years [19]. Humans have inhabited the continent for around 13,000 years [20], and *M*. *leprae* was the last to arrive, probably with exploration and colonization from Europe and human trafficking from Africa [21]. In Brazil, the first reported case of Hansen’s disease was in Rio de Janeiro in the 17th century [22]. It is reasonable to theorize that *M*. *leprae* was brought from Europe to Brazil and that, besides infecting other human beings, contaminated soil, water, and vegetation and, consequently, infected armadillos. Armadillos are uniquely susceptible to infection, harboring and multiplying the pathogen in their bodies and becoming an environmental source of the bacillus for humans. Eventually, when humans come into direct contact with the contaminated animal (or indirect contact via contaminated soil or water in the animal’s habitat), they can become infected and develop Hansen’s disease. People who develop the disease can multiply the bacillus and become a source of the bacteria for other individuals and the environment.

Although the 3 pillars of One Health—human, animal, and environmental—are evident in Brazil today in relation to Hansen’s disease, it remains to determine the strength of evidence for zoonotic transmission of *M*. *leprae* from wild armadillos to people in communities where hunting, handling, butchering, and eating armadillos are commonplace.

## Zoonotic Hansen’s disease in Brazil

Persons affected by Hansen’s disease in Brazil often report no known contact with another affected person, either in their household or outside. A study in Espírito Santo state from 2006 found that 55% of 506 patients had no known contact with infected individuals [23]. However, in the same group of patients, 68% reported direct contact (handling, butchering, or eating) with armadillos, compared with 48% of controls [24]. These data from Espírito Santo were combined in a recently published systematic review and meta-analysis with data from 2 other case–control studies, based in Ceará (2006) and Pará (2018), yielding a pooled odds ratio for Hansen’s disease of 2.23 (95% confidence interval [CI] 1.73, 2.88) comparing people who had direct armadillo contact with those in the same communities who did not have direct contact [25]. In the 3 studies, direct contact was defined as hunting or eating (Ceará); hunting, eating, or handling (Espírito Santo); and hunting (Pará, where it was noted that all cases who hunted also ate armadillo meat).

This systematic review found that the equivalent effect in the USA (also based on 3 case–control studies) was almost twice as high, with an odds ratio of 4.22 (95% CI 2.34, 7.59). This higher relative risk probably reflects the much lower background risk from person-to-person transmission in a non-endemic country. Other findings included an apparent dose–response relationship, from indirect contact (1.4-fold odds) to hunting armadillos (2.5-fold odds) [25].

The authors estimated that confounding and bias might reduce effect sizes by up to 40%, meaning that the additional zoonotic risk of Hansen’s disease for people in communities in Brazil where Hansen’s disease is endemic and contact with armadillos is common would be approximately 34% higher (with a CI ranging from a very small additional risk to 73% higher odds). The exact proportion of people in these communities who have direct contact with armadillos is unknown, but 34% higher odds yield population attributable fractions (the proportion of all cases of Hansen’s disease in the community attributable to contact with armadillos) of 3.3% (1 in 30 cases) if 10% of people have direct contact, 1 in 10 cases if 33% have direct contact, and 1 in 7 cases if half the people in the community have direct contact.

In endemic communities where person-to-person spread is the main mode of transmission, the additional risk of zoonotic transmission was evident in a cross-sectional study which reported a higher median anti-phenolic glycolipid 1 (PGL-1) titer in people who consumed armadillo meat more than once per month compared with not at all [26] and in a study among child and adolescent household contacts of Hansen’s disease cases which reported higher anti-natural octyl disaccharide-leprosy IDRI diagnostic (NDO-LID) antibody levels in those who had consumed armadillo meat compared to those who had not [27].

## One Health and armadillos

Armadillos are known to host a range of bacterial, fungal, protozoal, and other parasitic agents, including members of the genera *Histoplasma*, *Coccidioides*, *Trypanosoma*, *Toxoplasma*, *Sarcocystis*, *Leptospira*, *Sporothrix*, *Leishmania*, and *Paracoccidioides* [11,28,29]. Zoonotic transmission of these pathogens is unproven, but measures to reduce human contact with armadillos to prevent zoonotic transmission of *M*. *leprae* might reduce the incidence of other diseases. Potentially zoonotic viral infections in armadillos have not been described, but this almost certainly reflects a lack of investigation.

Interestingly, armadillos serve as a food source for the triatomine bugs (*Rhodnius prolixus*) that are vectors of *Trypanosoma cruzi*, the causative agent of Chagas disease, and as a reservoir of *T*. *cruzi* [29]. It has recently been demonstrated by experimental oral infection that *M*. *leprae* remain viable in the gut of *R*. *prolixus* for up to 20 days, move along the digestive tract, and that *M*. *leprae* from triatomine bug feces can be inoculated successfully into mouse foot pads [30]. However, studies of transmission of *M*. *leprae* to humans via arthropod vectors are mostly dated, and this hypothetical route requires investigation using molecular methods [16].

The other important aspect of armadillos in Brazil from a One Health perspective is their ecosystem role, which includes pest control, seed dispersal, nutrient cycling, and earth moving (for the benefit of other species) [11]. Protection of armadillo species in Brazil whether as a public health measure to prevent zoonotic Hansen and other human diseases or simply to ensure the survival of an endangered animal has consequences for ecosystems and biodiversity, thereby cementing the third pillar of a One Health approach.

## Applying a One Health approach to public health policy in Brazil

We have described the anomaly that is apparent in Hansen’s disease being recognized as a zoonotic disease in the USA, a non-endemic country where few people have contact with wild armadillos, but not in Brazil, an endemic country where contact with armadillos is common. We accept the prime role of person-to-person transmission in sustaining Hansen’s disease endemicity in Brazil, where the new case detection rate has not changed substantially over the past 10 years, from 20 new cases per 100,000 in 2009 to 14 per 100,000 in 2018 [10]. However, to ignore the contribution to Hansen’s disease endemicity of environmental sources and zoonotic transmission of *M*. *leprae* is negligent. It is also inconsistent with any long-term ambition to eliminate the disease. WHO Global Hansen’s Disease Strategy 2021–2030 stated that “eradication of [Hansen’s disease] is not feasible at this point of time due to presence of a zoonotic reservoir in some areas” and that “studies to understand the mode of zoonotic transmission and its overall epidemiological significance will be needed” [31]. Eradication (rather than elimination) is almost certainly not feasible at any point in time for environmental and sylvatic mycobacteria. However, the mention of zoonotic transmission is a welcome addition to global Hansen’s disease strategy, and we agree that research is needed, ideally directed from within endemic countries which have zoonotic reservoirs [32].

This research needs to be transdisciplinary, under the umbrella of One Health, encompassing social and anthropological research to understand community practices in relation to contact with wild armadillos [14], ecological studies in partnership with conservation groups to map and characterize transmission of *M*. *leprae* and its persistence within armadillos [11,12,29], and genomic studies to determine the relatedness of *M*. *leprae* strains infecting humans and animals [8,17]. A better understanding of pathways to infection, including that these might not be from person to person, is also important in destigmatising disease. Brazil has not only ample scientific capacity to conduct such research and build the necessary evidence base [4], but is also well placed to collaborate with neighboring countries where zoonotic transmission presents a barrier to the elimination of Hansen’s disease from endemic foci [32].

In the meantime, national programs to reduce endemicity and ultimately eliminate the disease from these countries should not ignore existing evidence for zoonotic transmission. From a One Health perspective, this approach is short sighted, both in terms of informing health professionals and the population about the risk of infection by *M*. *leprae* through contact with armadillos and in protecting the ecological richness of diverse and fragile ecosystems. The aim of this Viewpoint is to stimulate debate in Brazil, leading ultimately to a shift in public health policy. This is not a trivial task and will involve persuading many stakeholders including scientists and politicians. As a first step, we propose that public health authorities and medical associations in Brazil concerned with Hansen’s disease give serious consideration to updating guidelines in accordance with a One Health approach and consistent with WHO Global Hansen’s Disease Strategy 2021–2030.
